# Advancing Zebrafish (*Danio rerio*) Welfare Using Immersion Analgesics

**DOI:** 10.3390/vetsci12060571

**Published:** 2025-06-11

**Authors:** Cláudia A. Rocha, Luís M. Félix, Sandra M. Monteiro, Carlos Venâncio

**Affiliations:** 1 Centre for the Research and Technology of Agroenvironmental and Biological Sciences (CITAB, Inov4Agro), Universidade de Trás-os-Montes e Alto Douro (UTAD), Quinta de Prados, 5000-801 Vila Real, Portugal; xana3@live.com.pt (C.A.R.); lfelix@utad.pt (L.M.F.); smonteir@utad.pt (S.M.M.); 2 Institute for Innovation, Capacity Building and Sustainability of Agri-Food Production (Inov4Agro), UTAD, Quinta de Prados, 5000-801 Vila Real, Portugal; 3 Department of Biology and Environment, School of Life and Environmental Sciences, UTAD, Quinta de Prados, 5000-801 Vila Real, Portugal; 4 Department of Animal Science, School of Agrarian and Veterinary Sciences (ECAV), UTAD, Quinta de Prados, 5000-801 Vila Real, Portugal

**Keywords:** analgesia, behavior, immersion, nociception, pain, zebrafish

## Abstract

**Simple Summary:**

Evidence of pain perception in fish continuously raises concerns regarding their welfare. Despite this, the field of fish analgesia remains underdeveloped and lacks effective non-invasive solutions. The widespread use of zebrafish as a model organism exposes them to a variety of potentially painful procedures, placing them at the forefront of these concerns. This review summarizes the existing data on immersion analgesics by analyzing behavioral indicators of nociception. Although some promising candidates—opioids, NSAIDs, a local anesthetic, and natural monoterpenes—were identified, further research is needed to assess additional effects associated with their administration. These findings highlight the importance of selecting appropriate analgesics according to the duration and severity of the noxious stimulus, as well as the need for detailed water quality monitoring and parameter reporting to safeguard fish welfare. As research progresses, extending these insights beyond zebrafish is essential to improve welfare standards in a broader context.

**Abstract:**

Growing evidence of pain perception in fish has raised concerns about their welfare in different contexts, including research and aquaculture, where potentially painful procedures are standard. Despite ongoing efforts to advance fish analgesia, the field remains underdeveloped, particularly regarding less invasive methods that could improve welfare, such as immersion-based analgesia. As one of the most widely used models in research, zebrafish (*Danio rerio*) are often at the front of these concerns. This review aimed to consolidate the current knowledge on immersion analgesics by analyzing the behavioral responses of zebrafish exposed to noxious stimuli. Some promising immersion analgesics were identified; however, further research is needed to assess their effects on additional parameters and investigate potential adverse effects. These findings underscore the importance of selecting appropriate analgesics, as their effectiveness may vary depending on the duration and severity of the stimulus. Moreover, this review highlights the critical role of consistent water quality monitoring and detailed parameter reporting, as these factors may influence analgesic efficacy and compromise fish welfare. As research advances, applying these insights beyond zebrafish to other fish species will be crucial for promoting higher welfare standards.

## 1. Introduction

**Figure 1:**
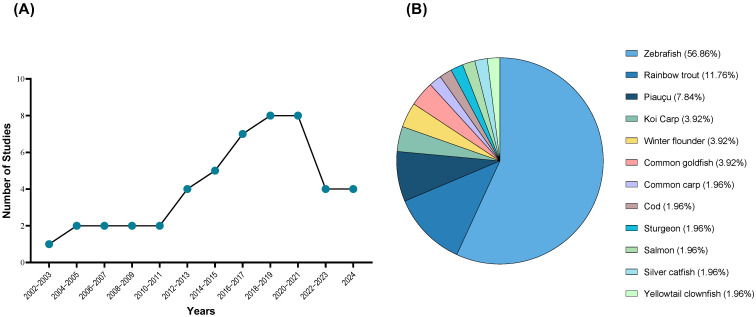
Progress in fish analgesia research (**A**) and the species used for studying prospective analgesics (**B**) following the recognition of fish nociception in 2002. These studies (49) were retrieved after a database (PubMed, PubMedCentral, Scopus, Europe PMC, and Web of Science) search using the keywords “fish”, “analgesia”, “antinociception”, “pain”, and “nociception.” As some studies used more than one species, the total percentage was calculated based on the number of species mentioned (n = 51).

Growing evidence consistently demonstrates that fish feel pain, raising concerns about their welfare, particularly in settings where potentially painful procedures might be implemented [1,2,3,4,5]. Despite rising awareness, research on analgesic solutions remains limited, and a definitive approach to addressing this issue is still lacking (Figure 1A).

Nociception is the physiological process by which nociceptors detect noxious stimuli, usually triggering a reflex withdrawal response [2,5]. It was first identified in rainbow trout (*Oncorhynchus mykiss*) in 2002 [6] and has since been described in other teleost species such as winter flounder (*Pseudopleuronectes americanus*) [7,8], common goldfish (*Carassius auratus*) [8,9], zebrafish (*Danio rerio*) [10,11], Atlantic salmon (*Salmo salar*) [12], and silver catfish (*Rhamdia quelen*) [13] (Figure 1B). Although all animals possess nociception and can reflexively respond to harmful stimuli, this does not necessarily equate to experiencing pain [14]. Pain perception involves a psychological component and higher processing of the noxious stimuli, which raises questions about whether fish are capable of such higher processing [14,15]. However, indicators of pain perception have been demonstrated in multiple studies, challenging the uncertainty and confirming that fish are indeed capable of experiencing pain [2,15,16,17,18].

Zebrafish (*Danio rerio*) have become one of the most widely used models in scientific research and are among the most species used in the field of analgesia due to their high reproductive capacity, external and rapid larval development, and ease of manipulation, which offers considerable advantages across multiple research areas [5,19,20]. However, alongside their increasing use, concerns about zebrafish welfare are also growing, highlighting the urgent need for effective solutions to minimize their discomfort [21,22,23].

Over the years, zebrafish have also emerged as a key model in advancing the understanding of nociception, from identifying its molecular mechanisms to exploring novel substances that could refine painful procedures and improve fish welfare [5,22,24,25]. Transient receptor potential (TRP) channels, such as cation-permeable TRP vanilloid type-1 (TRPV1) and TRP ankyrin1 (TRPA1) channels, are essential to the process of nociception due to their central role in meditating the detection of noxious stimuli [24,25]. While TRPA1 is specific for detecting chemical noxious stimuli, TRPV1 responds to a broader range of stimuli, including high temperatures, low pH, and specific chemical agents [24,25,26,27]. Multiple stimuli effectively activate these receptors, resulting in alterations in behavior that ultimately reflect noxious responses [3,8,28,29,30]. These behavioral alterations not only indicate nociception but also suggest pain, thus contributing to the investigation of potential analgesic substances by assessing their ability to reverse these changes and minimize discomfort [3,31,32,33]. Although the information on analgesic substances for fish species is still limited, drugs targeting TRPA1 or TRPV1 channels present promising candidates for novel analgesic development [24,25,26,27].

**Figure 2:**
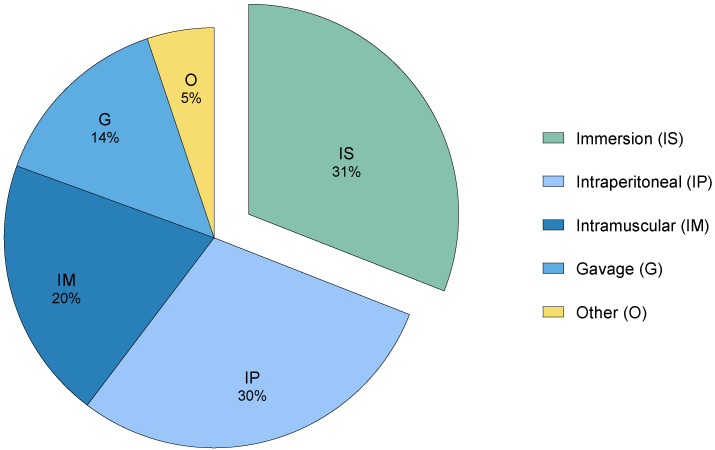
The distribution of different analgesic administration methods in research (2002–2024), categorized as invasive and non-invasive. These studies (41) were retrieved after a database (PubMed, PubMedCentral, Scopus, Europe PMC, and Web of Science) search using the keywords “fish”, “analgesia”, “antinociception”, “pain”, and “nociception”.

In recent decades, several analgesics have been considered as suitable options for zebrafish, including conventional mammalian analgesics like opioids [3,11,13,34], non-steroidal anti-inflammatory drugs (NSAIDs) [26,29,35], and local anesthetics [28,36], as well as other plant-based substances [37,38,39,40]. However, the effects and potential side effects of these substances remain poorly documented [22]. In addition, their administration often relies on invasive methods (Figure 2), which can be particularly challenging for a small species like zebrafish due to handling and injection [5,41].

Immersion analgesics have been studied as a potential solution to this challenge. However, despite being less invasive, their effectiveness still depends on each substance’s pharmacokinetic properties, which can challenge its efficacy by limiting adsorption, metabolism, and elimination [42]. Nevertheless, exploring immersion analgesics could offer a less invasive approach and contribute to the overall well-being of zebrafish. Due to the critical role of zebrafish as a model organism in research, this review aims to consolidate the existing literature on zebrafish analgesia and evaluate the efficacy of immersion analgesics by analyzing behavioral data. Through this, the review aims to provide a comprehensive overview of the current state of the field, highlighting the existing knowledge gaps and addressing the challenges that must be overcome to advance zebrafish welfare.

## 2. Materials and Methods

### 2.1. Database Search

The main goal of this review was to gather the most up-to-date scientific evidence regarding analgesic solutions in zebrafish. To achieve this, a database search was conducted, following the Preferred Reporting Items for Systematic Reviews and Meta-Analysis (PRISMA) protocol [43], to identify relevant information published from the acknowledgement of nociception in fish [6] up to December 2024, using “PubMed”, “PubMedCentral”, “Scopus”, “Europe PMC”, and “Web of Science”. Boolean connectors (AND, OR) were used to search for the keywords “zebrafish”, “*Danio rerio*”, “analgesia”, “antinociception”, “pain”, and “nociception” across the five databases.

### 2.2. Eligibility Criteria

The studies included in this review were (i) novel, non-retracted, peer-reviewed research papers that (ii) used zebrafish as biological research models, (iii) involved the immersion in an analgesic substance after (iv) exposure to a noxious stimulus, and (v) assessed the same behavioral outcomes. These outcomes were selected based on the availability of studies evaluating the same behavior, allowing for appropriate grouping. In addition, the papers needed to include (vii) the life stage of the animal models, (viii) the sampling distributions (mean, standard deviation (SD), and sample size (n)) of the treated groups, as well as (viii) the temperature and pH of the water, as these parameters might influence the experimental results [44]. Despite dissolved oxygen levels being a fundamental parameter to ensure optimal conditions for fish, this factor is often overlooked in most studies and was not considered an inclusion criterion in this review [45].

Excluded studies consisted of (i) those without full-text available, (ii) non-English papers, (iii) published before 2002, (iv) reviews, abstracts, or books, (v) studies unrelated to the topic or with missing data, and (vi) those that did not observe analgesic effects up to 30 min after exposure to the noxious stimulus to ensure consistency across the data. Additionally, (viii) studies involving the administration of an anesthetic either before or after the analgesic were excluded to avoid potential interference from the anesthetic on the observed results [46].

### 2.3. Data Extraction and Organization

The search results from the database were exported into EndNote, where duplicates and papers published before 2002 were removed [47], and the remaining studies were screened based on titles and abstracts, with relevant papers undergoing further analysis. The references of the selected papers were reviewed to identify any additional relevant publications.

Data from the eligible studies, such as the first author and year of publication; analgesic substance and its concentration; life stage and sample size; water temperature (°C), and pH; noxious stimulus and respective behavioral responses, were organized based on the behavior parameter observed and the nature of the noxious stimulus (chemical or thermal) used. All substance concentrations were standardized to a uniform unit (mg/L) to ensure consistency across the dataset.

When behavioral data were presented solely in graphical format, values were extracted using WebPlot Digitizer for analysis. The mean and SD were calculated directly when the standard error of the mean was provided or estimated from median and interquartile values using the method described by McGrath et al. (2020) [48] (accessible online at: https://smcgrath.shinyapps.io/estmeansd/; accessed on 4 September 2024). The retrieved data were normalized to their respective control values to reduce potential bias from varying baseline measurements and facilitate meaningful comparisons [26].

One author (CR) screened the retrieved literature and cross-checked the data according to the inclusion and exclusion criteria. Any questions or uncertainties were resolved with the assistance of another author (LF, SMM, or CV).

## 3. Results

### 3.1. Study Selection

**Figure 3:**
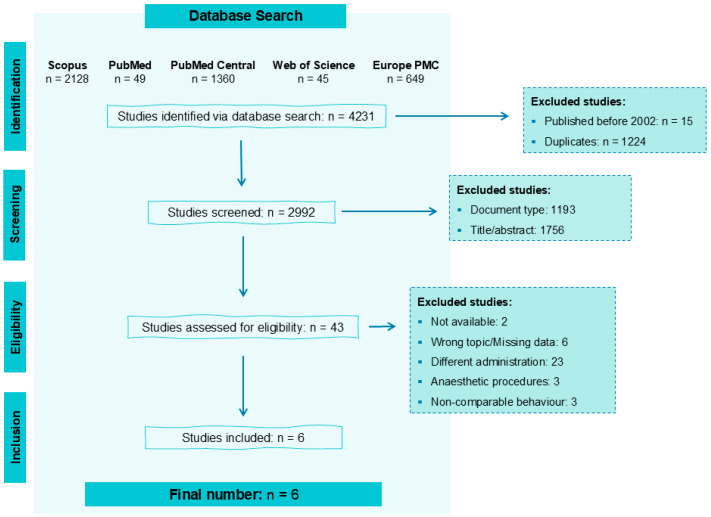
Workflow for the identification of studies to be included in this review, indicating the reasons for exclusion and the number of articles in each category.

A total of 4231 papers were identified from the selected databases: 2128 from Scopus, 49 from PubMed, 1360 from PubMed Central, 45 from Web of Science, and 649 from Europe PMC (Figure 3).

Among these, 15 were published before 2002, and 1224 were duplicates, leaving 2992 articles for assessment. After initial screening based on document type, title, and abstract, 2949 papers were excluded, and 43 were assessed for eligibility. From these, 6 articles were to be included in the review. An additional bibliographic search on these studies was conducted, but no additional papers were selected.

### 3.2. Study Characteristics

The selected studies used zebrafish distributed between early larval stages (83%) with 5 days post-fertilization (dpf) and late larval stages (17%) with 12–14 dpf, and the number of animals used per treatment ranged from 14 to 450. Water parameters in the zebrafish enclosures were maintained within optimal ranges [49], with temperature between 28 and 28.5 °C and pH from 7.2 to 7.5. The studies reported the effects of nine potential analgesic substances, including lidocaine, described in four articles, morphine in three articles, and flunixin, aspirin, and fentanyl, each covered in one article. Additionally, one study explored four natural substances: eugenol, menthol, thymol, and carvacrol. Regarding behavioral responses, two studies reported the substance’s effect on velocity (mm/s), two on the percentage of active time, and two reported on both velocity and percentage of active time. Normalized swimming velocity (Table 1) after a chemical stimulus varied from 0.76 [40] to 2.31 mm/s [50], while normalized swimming velocity after a thermal stimulus ranged between 0.97 and 1.01 mm/s [29].

Similarly, normalized active time (Table 2) following a chemical stimulus ranged from 0.86 to 1.02% [51,52], while after a thermal stimulus, it varied from 1.10% to 1.22% [29].

## 4. Discussion

**Figure 4:**
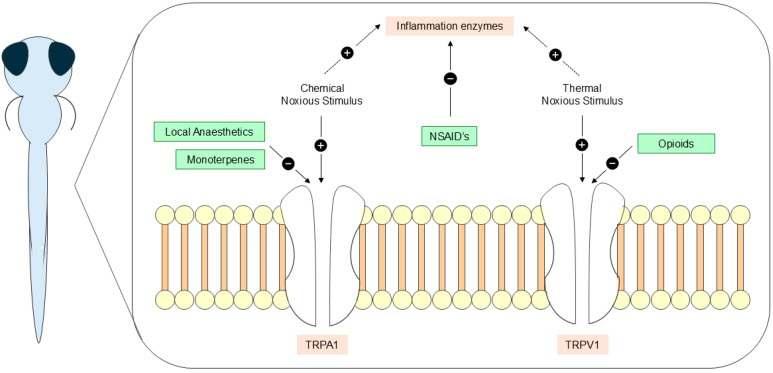
Local anesthetics, monoterpenes, NSAIDs, and opioid analgesics potential mechanisms of action in zebrafish (*Danio rerio*) larval stages.

Over the past decades, numerous studies have provided evidence that fish experience pain [2,3]. However, despite the increasing use of zebrafish as a model organism, research on analgesic substances for this species remains limited [1,22]. Behavioral responses serve as important indicators of nociception and pain as they represent immediate and easily observable measures [53]; but, assessing the efficacy of analgesics across studies presents challenges since some parameters, like active time, may decrease [33], while others, like swimming velocity, can either increase [26] or decrease [40] in response to noxious stimuli. Although behavioral endpoints are valuable, complementing these assessments with physiological and molecular indicators of nociception is fundamental for a more comprehensive understanding of analgesic and pain responses [5,53]. Nevertheless, this review identified some potential substances to use as immersion analgesics, including opioids morphine [29,36,52] and fentanyl [50], NSAID’s aspirin [52] and flunixin [29], local anesthetic lidocaine [29,36,51,52], and natural monoterpenes eugenol, menthol, carvacrol, and thymol [40]. Most of these substances revealed beneficial effects in response to different noxious stimuli, accentuating their role as candidates for fish analgesia and highlighting their potential mechanisms of action (Figure 4).

In terms of swimming velocity, thymol effectively reversed the chemical nociception induced by 0.05% acetic acid, while lidocaine revealed similar effects at 0.1% acetic acid. Acetic acid is a widely used nociceptive agent in aquatic species, making it a common tool for assessing the efficacy of analgesic substances [5]. Its administration in water decreases pH, leading to intracellular acidification and activation of TRPA1 channels that are responsible for modulating the response to noxious stimuli by regulating the cation influx into sensory neurons [2,25,54,55]. Natural monoterpene thymol shows its antinociceptive effects through multiple mechanisms, which potentially include modulation of sodium (Na^+^) and potassium (K^+^) channels [56]. Similarly, lidocaine—a well-established local anesthetic—interacts with cation channels, such as K^+^, Na^+^, and calcium (Ca^2+^), reducing the influx of ions entering neurons and their potential to transmit nociceptive signals [5,22,57]. Through modulation of these channels, thymol and lidocaine might act as antagonists of TRPA1, which justifies their capacity to reverse the effects of a noxious chemical stimulus. However, while these substances show promise, their safety profile in zebrafish remains unclear and should be the focus of future investigation. In addition, they exhibit notable differences: while lidocaine is a well-established analgesic, thymol has yet to be fully recognized as one, as research on the antinociceptive effects of monoterpenes is still in its early stages. Nevertheless, interest in plant-based substances like monoterpenes is growing as they are being widely studied for their potentially lower risk of adverse effects, offering a safer alternative to conventional analgesics [37,38,39,40]. Still, as these substances exhibit their antinociceptive potential through complex interactions with various systems, their use could have unwanted effects on other parameters, warranting further investigation [56].

Although most substances demonstrate analgesic activity, it is important to discuss the increase in swimming velocity following fentanyl exposure [50]. Fentanyl is an effective opioid analgesic known for its rapid effect in humans [58], reinforcing its potential as an effective analgesic for fish. However, the observed increase in swimming velocity when fentanyl is combined with formalin raises questions regarding its effects. Formalin is a popular noxious stimulus due to its ability to induce nociception in two distinct phases- neurogenic and inflammatory, making it a valuable tool for assessing analgesic efficacy [59,60]. During the first five minutes of formalin nociception, this stimulus activates TRPA1 channels, initiating a neurogenic response that leads to tissue damage, followed by an inflammatory phase mediated by prostaglandins [33,54,60,61]. Because of this, formalin induces a more severe and prolonged nociceptive response compared to other noxious stimuli [60]. In addition, in Zaig et al. (2021) [50], zebrafish were exposed to formalin in combination with fentanyl for 15 min, whereas in the studies by Rocha et al. (2024) [40] and Lopez-Luna et al. (2017a) [29], there was a shorter exposure time of just 1 min. The discrepancy in exposure duration, combined with the severity of the stimulus, might explain the increased swimming velocity observed, rather than indicating an understatement of fentanyl’s efficacy. Still, when fentanyl was combined with AITC, there was also a slight increment in swimming velocity. AITC induces nociception by activating TRPA1 channels through direct modification of the channel’s cysteine residues, which, in turn, triggers an inflammatory response [62,63]. However, AITC also leads to rapid desensitization and internalization of TRPA1 channels, resulting in reduced noxious stimulus signaling over time [54]. This desensitization effect may contribute to the observed increase in swimming velocity, as the nociceptive response diminishes with prolonged exposure to AITC. Despite the prolonged exposure, AITC’s ability to induce nociception consequently decreases, contrasting with the persistent response exhibited by formalin. The differences observed with these two noxious stimuli suggest that fentanyl’s efficacy might vary depending on the severity and duration of the stimulus, accentuating the importance of selecting analgesics based on the stimulus to which the animal is exposed. Nevertheless, additional studies are needed to fully understand the role of fentanyl in fish analgesia. These investigations should focus on determining its long-term effects, optimal dosages, and safety profile in zebrafish, as well as exploring the potential for fentanyl to be used in combination with other analgesic agents to enhance its efficacy and reduce any adverse effects. Further research will be crucial for validating fentanyl’s therapeutic potential and for establishing evidence-based protocols for its use in fish welfare management.

Against thermal nociception, both lidocaine and morphine emerged as potentially effective analgesics, with morphine standing out as the preferred option, as its results in swimming velocity align with active time. The increase in water temperature to 40 °C activates TRPV1 receptors, which act similarly to TRPA1 in the process of nociception [24,25]. Morphine is an opioid drug that acts on µ, δ, or κ opioid receptors in the brain, inhibiting the release of neurotransmitters and blocking the activity of nociceptors, thereby altering pain perception [6,22]. Although limited studies report morphine’s antinociceptive mechanism in fish, evidence suggests that morphine also modulates TRPV1 receptors, which could explain its effectiveness against thermal noxious stimuli [64]. Morphine also proved to be effective against 10% soda water, a chemical noxious stimulus that activates TRPA1 receptors, indicating that multiple mechanisms may contribute to its powerful analgesia [25,36]. Soda water increases carbon dioxide (CO_2_) concentration, which not only activates nociceptive pathways but also lowers the water pH and, at higher concentrations, has the potential to induce anesthesia, potentially influencing behavioral responses and highlighting the complexity of interpreting analgesic efficacy under such conditions [25,36]. Studies on the use of opioid drugs in fish evidence their pronounced adverse effects, especially on the cardiorespiratory system, raising concerns about their long-term viability as analgesics [22,50,65]. In addition, the restrictive regulations and high costs of opioid drugs make their use for fish analgesia less viable, urging the need to find novel, effective, and safer alternatives [28,66]. In response, lidocaine presents an interesting solution since it demonstrated consistent results against 10% soda water and 0.1% acetic acid, further strengthening its role as a TRPA1 antagonist. Despite these advancements, the lack of established indicators of pain in fish, which arises from the limited knowledge of their physiology and behavior [22], complicates the field of analgesia, underscoring the need for further studies to complement ongoing research on nociception. Although the limited number of studies fitting the established criteria also stands as a limitation to advancing fish analgesia, comparing the existing data remains valuable to guide future research.

Among the tested substances, flunixin was the only one that demonstrated ambiguous results, improving active time but having no influence on velocity. Flunixin is an NSAID whose mechanism of action relies on the inhibition of enzymes, such as cyclooxygenase (COX), that are involved in the inflammatory process [67,68]. Because of this, the use of these types of drugs is typically associated with inflammation arising from painful responses [67,68]. In Lopez-Luna et al. (2017a) [29], zebrafish larvae were exposed for 1 min to water at 40 °C, a noxious stimulus that simulates acute pain and which may not progress to inflammatory pain due to its short duration [28,29,36,52,69,70]. As such, the inflammatory enzymes that flunixin acts on are not released, which potentially justifies its questionable results. This analgesic might still represent a promising candidate for fish analgesia; however, before it can be considered reliable, additional studies must be conducted, including prolonged exposure to noxious stimuli or stimuli known to induce inflammation. Moreover, despite the effects of NSAIDs not being commonly investigated, ecotoxicological studies have identified potential adverse effects following chronic administration, including nephrotoxicity and hepatoxicity, which could question their applicability in the field [22].

Selecting effective analgesics is important to improve fish welfare. However, other factors, such as water quality maintenance, must also be considered to safeguard both welfare and the integrity of experimental results, since poor water quality can affect the behavior and physiology of fish [71,72]. While the water temperature and pH in the enclosures of the studies reviewed here were within the optimal range for zebrafish housing [49], a major limitation was the frequent omission of dissolved oxygen levels. This parameter is critical to monitor since its levels can fluctuate in response to changes in temperature, pH, alkalinity, and chemical agents added to the water [45,71,73,74], contributing to a stressful environment for the fish and potentially influencing analgesic efficacy [45,75]. This lack of information highlights the importance of proper monitoring and reporting of the conditions in which animals are kept, to avoid confounding factors that could impact the welfare of the fish and the reliability of the experimental findings. In addition, it is also important to acknowledge potential differences in drug uptake between larval and adult zebrafish stages, as most data on immersion analgesia involves larvae. Future studies should aim to expand the range of tested analgesics while conducting a more in-depth investigation across different noxious stimuli, with the possibility of establishing standardized protocols for assessing analgesic efficacy being of immense value. Alongside this, continued research on zebrafish nociceptive mechanisms remains equally important, especially to understand how environmental parameters and development stages might influence drug uptake and effectiveness.

## 5. Conclusions

Research in the field of fish analgesia has significantly advanced over the past decade, yet there is still a long way to go before safe and effective analgesics for zebrafish are fully implemented. This review identified some promising candidates that demonstrated effectiveness in counteracting the noxious stimuli. However, these substances warrant additional investigation to thoroughly assess their impact on other relevant parameters and to evaluate their potential adverse effects before they can be adopted as standard analgesics for zebrafish. Notably, this review emphasizes the importance of selecting appropriate analgesics since their efficacy might vary depending on the severity and duration of the noxious stimuli. Furthermore, it is necessary to maintain and report the water conditions in fish enclosures to ensure the results on analgesia are not influenced by these factors and that no additional stress is involved. In the future, additional analgesics must be screened and more thorough investigations across different noxious stimuli must be conducted, possibly leading to the establishment of standardized protocols for assessing analgesic efficacy. Alongside this, research into the mechanisms of zebrafish nociception must continue with the aim of better understanding how external factors and developmental stages might impact drug uptake and effectiveness. As research progresses, it will be important to conduct more studies focusing on immersion analgesics not only to identify the most suitable option for zebrafish but also to apply these findings to other fish species, thus advancing welfare across a broader range of species.

## Figures and Tables

**Table 1:** Potential immersion analgesics identified by analyzing the swimming velocity (mm/s) of zebrafish (*Danio rerio*) ^1^.

Noxious Stimulus		Zebrafish Stage	Compound	Concentration (mg/L)	Normalized Swimming Velocity	Reference
Chemical	Acetic acid (0.05%)	5 dpf	Eugenol	10	0.76	[40]
	Acetic acid (0.05%)	5 dpf	Menthol	2	1.11	[40]
	Acetic acid (0.05%)	5 dpf	Thymol	2	1.04	[40]
	Acetic acid (0.05%)	5 dpf	Carvacrol	2	1.14	[40]
	Acetic acid (0.1%)	5 dpf	Lidocaine	5	0.91	[51]
	Formalin (0.05%)	12–14 dpf	Fentanyl	1	2.31	[50]
	Allyl isothiocyanate (AITC) (100 uM)	12–14 dpf	Fentanyl	2	1.28	[50]
Thermal	Hot water (40 °C)	5 dpf	Morphine	48	1.01	[29]
	Hot water (40 °C)	5 dpf	Lidocaine	5	0.97	[29]

^1^ Data was normalized according to their respective control groups ([50]: fentanyl (1 mg/L): 0.45 mm/s, fentanyl (2 mg/L): 0.64 mm/s; [51]: 2.32 mm/s; [40]: 10.82 mm/s; [29]: 2.37 mm/s).

**Table 2:** Potential immersion analgesics identified by analyzing the percentage (%) of zebrafish (*Danio rerio*) active time ^2^.

Noxious Stimulus		Zebrafish Stage	Compound	Concentration (mg/L)	Normalized % Active Time	Reference
Chemical	Acetic acid (0.1%)	5 dpf	Lidocaine	5	0.86	[51]
	Acetic acid (0.1%)	5 dpf	Lidocaine	5	1.02	[52]
	Acetic acid (0.1%)	5 dpf	Morphine	48	0.86	[52]
	Acetic acid (0.1%)	5 dpf	Aspirin	2.5	0.88	[52]
	Soda water (10%)	5 dpf	Lidocaine	5	0.96	[36]
	Soda water (10%)	5 dpf	Morphine	48	1.00	[36]
Thermal	Hot water (40 °C)	5 dpf	Flunixin	20	1.15	[29]
	Hot water (40 °C)	5 dpf	Lidocaine	5	1.22	[29]
	Hot water (40 °C)	5 dpf	Morphine	48	1.10	[29]

^2^ Data was normalized according to their respective control groups ([51]: 66.41%; [52]: 71.02%; [36]: 75.42%; [29]: 60.28%).

## Data Availability

The data supporting the findings of this article will be provided by the authors upon request.

## Abbreviations

The following abbreviations are used in this manuscript:

TRP	Transient Receptor Potential
TRPV1	Transient Receptor Potential Vanilloid Type-1
TRPA1	Transient Receptor Potential Ankyrin1
NSAIDs	Nonsteroidal anti-inflammatory drugs
AITC	Allyl-isothiocyanate
dpf	Days Post Fertilization
Na^+^	Sodium
K^+^	Potassium
Ca^2+^	Calcium
CO_2_	Carbon dioxide
COX	Cyclooxygenase

## References

[1] Martins T., Valentim A., Pereira N., Antunes L.M. (2019). Anaesthetics and analgesics used in adult fish for research: A review. Lab. Anim..

[2] Sneddon L.U. (2019). Evolution of nociception and pain: Evidence from fish models. Philos. Trans. R. Soc. Lond. B Biol. Sci..

[3] Sneddon L.U. (2003). The evidence for pain in fish: The use of morphine as an analgesic. Appl. Anim. Behav. Sci..

[4] Sneddon L.U. (2003). Trigeminal somatosensory innervation of the head of a teleost fish with particular reference to nociception. Brain Res..

[5] Ohnesorge N., Heinl C., Lewejohann L. (2021). Current Methods to Investigate Nociception and Pain in Zebrafish. Front. Neurosci..

[6] Sneddon L.U. (2002). Anatomical and electrophysiological analysis of the trigeminal nerve in a teleost fish, *Oncorhynchus mykiss*. Neurosci. Lett..

[7] Newby N.C., Gamperl A.K., Stevens E.D. (2007). Cardiorespiratory effects and efficacy of morphine sulfate in winter flounder (*Pseudopleuronectes americanus*). Am. J. Vet. Res..

[8] Newby N.C., Wilkie M.P., Stevens E.D. (2009). Morphine uptake, disposition, and analgesic efficacy in the common goldfish (*Carassius auratus*). Can J. Zool..

[9] Nordgreen J., Garner J.P., Janczak A.M., Ranheim B., Muir W.M., Horsberg T.E. (2009). Thermonociception in fish: Effects of two different doses of morphine on thermal threshold and post-test behaviour in goldfish (*Carassius auratus*). Appl. Anim. Behav. Sci..

[10] Correia A.D., Cunha S.R., Scholze M., Stevens E.D. (2011). A Novel Behavioral Fish Model of Nociception for Testing Analgesics. Pharmaceuticals.

[11] Steenbergen P.J., Bardine N. (2014). Antinociceptive effects of buprenorphine in zebrafish larvae: An alternative for rodent models to study pain and nociception?. Appl. Anim. Behav. Sci..

[12] Nordgreen J., Bjørge M.H., Janczak A.M., Poppe T., Koppang E.O., Ranheim B., Horsberg T.E. (2013). The effect of morphine on changes in behaviour and physiology in intraperitoneally vaccinated Atlantic salmon (*Salmo salar*). Appl. Anim. Behav. Sci..

[13] Rodrigues P., Barbosa L.B., Bianchini A.E., Ferrari F.T., Baldisserotto B., Heinzmann B.M. (2019). Nociceptive-like behavior and analgesia in silver catfish (*Rhamdia quelen*). Physiol. Behav..

[14] Raja S.N., Carr D.B., Cohen M., Finnerup N.B., Flor H., Gibson S., Keefe F.J., Mogil J.S., Ringkamp M., Sluka K.A. (2020). The revised International Association for the Study of Pain definition of pain: Concepts, challenges, and compromises. Pain.

[15] Sneddon L.U. (2006). Ethics and welfare: Pain perception in fish. Bull. Eur. Ass. Fish Pathol..

[16] Dunlop R., Laming P. (2005). Mechanoreceptive and nociceptive responses in the central nervous system of goldfish (*Carassius auratus*) and trout (*Oncorhynchus mykiss*). J. Pain.

[17] Sneddon L.U., Schroeder P., Roque A., Finger-Baier K., Fleming A., Tinman S., Collet B. (2024). Pain management in zebrafish: Report from a FELASA Working Group. Lab. Anim..

[18] Sneddon L.U., Braithwaite V.A., Gentle M.J. (2003). Novel object test: Examining nociception and fear in the rainbow trout. J. Pain.

[19] Briggs P.J. (2002). The zebrafish: A new model organism for integrative physiology. Am. J. Physiol..

[20] Meyers J.R. (2018). Zebrafish: Development of a Vertebrate Model Organism. Curr. Protoc. Essent. Lab. Tech..

[21] Weber E.S. (2011). Fish analgesia: Pain, stress, fear aversion, or nociception?. Vet. Clin. N. Am. Exot. Anim. Pract..

[22] Chatigny F., Creighton C.M., Stevens D. (2018). Updated Review of Fish Analgesia. J. Am. Assoc. Lab. Anim..

[23] Harms C. (2005). Surgery in Fish Research: Common Procedures and Postoperative Care. Lab. Anim..

[24] Palazzo E., Rossi F., Maione S. (2008). Role of TRPV1 receptors in descending modulation of pain. Mol. Cell. Endocrinol..

[25] Wang Y.Y., Chang R.B., Allgood S.D., Silver W.L., Liman E.R. (2011). A TRPA1-dependent mechanism for the pungent sensation of weak acids. J. Gen. Physiol..

[26] Curtright A., Rosser M., Goh S., Keown B., Wagner E., Sharifi J., Raible D.W., Dhaka A. (2015). Modeling nociception in zebrafish: A way forward for unbiased analgesic discovery. PLoS ONE.

[27] Prober D.A., Zimmerman S., Myers B.R., McDermott B.M., Kim S.H., Caron S., Rihel J., Solnica-Krezel L., Julius D., Hudspeth A.J. (2008). Zebrafish TRPA1 channels are required for chemosensation but not for thermosensation or mechanosensory hair cell function. J. Neurosci..

[28] Deakin A.G., Buckley J., AlZu’bi H.S., Cossins A.R., Spencer J.W., Al’Nuaimy W., Young I.S., Thomson J.S., Sneddon L.U. (2019). Automated monitoring of behaviour in zebrafish after invasive procedures. Sci. Rep..

[29] Lopez-Luna J., Al-Jubouri Q., Al-Nuaimy W., Sneddon L.U. (2017). Impact of analgesic drugs on the behavioural responses of larval zebrafish to potentially noxious temperatures. Appl. Anim. Behav. Sci..

[30] Steenbergen P.J. (2018). Response of zebrafish larvae to mild electrical stimuli: A 96-well setup for behavioural screening. J. Neurosci. Methods.

[31] Reilly S.C., Quinn J.P., Cossins A.R., Sneddon L.U. (2008). Behavioural analysis of a nociceptive event in fish: Comparisons between three species demonstrate specific responses. Appl. Anim. Behav. Sci..

[32] Kalueff A.V., Gebhardt M., Stewart A.M., Cachat J.M., Brimmer M., Chawla J.S., Craddock C., Kyzar E.J., Roth A., Landsman S. (2013). Towards a Comprehensive Catalog of Zebrafish Behavior 1.0 and Beyond. Zebrafish.

[33] Magalhães F.E.A., de Sousa C., Santos S., Menezes R.B., Batista F.L.A., Abreu A.O., de Oliveira M.V., Moura L., Raposo R.D.S., Campos A.R. (2017). Adult Zebrafish (*Danio rerio*): An Alternative Behavioral Model of Formalin-Induced Nociception. Zebrafish.

[34] Baker T.R., Baker B.B., Johnson S.M., Sladky K.K. (2013). Comparative analgesic efficacy of morphine sulfate and butorphanol tartrate in koi (*Cyprinus carpio*) undergoing unilateral gonadectomy. J. Am. Vet. Med. Assoc..

[35] Mettam J.J., Oulton L.J., McCrohan C.R., Sneddon L.U. (2011). The efficacy of three types of analgesic drugs in reducing pain in the rainbow trout, *Oncorhynchus mykiss*. Appl. Anim. Behav. Sci..

[36] Lopez-Luna J., Canty M.N., Al-Jubouri Q., Al-Nuaimy W., Sneddon L.U. (2017). Behavioural responses of fish larvae modulated by analgesic drugs after a stress exposure. Appl. Anim. Behav. Sci..

[37] Correia A.M., Pedrazzani A.S., Mendonca R.C., Massucatto A., Ozorio R.A., Tsuzuki M.Y. (2018). Basil, tea tree and clove essential oils as analgesics and anaesthetics in *Amphiprion clarkii* (Bennett, 1830). Braz. J. Biol..

[38] Soares I.C.R., Santos S.A.A.R., Coelho R.F., Alves Y.A., Vieira-Neto A.E., Tavares K.C.S., Magalhaes F.E.A., Campos A.R. (2019). Oleanolic acid promotes orofacial antinociception in adult zebrafish (*Danio rerio*) through TRPV1 receptors. Chem. Biol. Interact..

[39] Pereira W.F., Everson da Silva L., do Amaral W., Andrade Rebelo R., Quefi B., Wlisses da Silva A., Silva Marinho E., Borges Leal A.L.A., Mesquita Cajazeiras F.F., Amancio Ferreira M.K. (2024). Essential Oils from the Genus Piper Promote Antinociception by Modulating TRP Channels and Anti-Inflammatory Effects in Adult Zebrafish. Chem. Biodivers..

[40] Rocha C.A., Felix L.M., Monteiro S.M., Venancio C. (2024). Antinociceptive Analysis of Natural Monoterpenes Eugenol, Menthol, Carvacrol and Thymol in a Zebrafish Larval Model. Pharmaceuticals.

[41] Schroeder P.G., Sneddon L.U. (2017). Exploring the efficacy of immersion analgesics in zebrafish using an integrative approach. Appl. Anim. Behav. Sci..

[42] Zahl I.H., Samuelsen O., Kiessling A. (2012). Anaesthesia of farmed fish: Implications for welfare. Fish Physiol. Biochem..

[43] Page M.J., McKenzie J.E., Bossuyt P.M., Boutron I., Hoffmann T.C., Mulrow C.D., Shamseer L., Tetzlaff J.M., Akl E.A., Brennan S.E. (2021). The PRISMA 2020 statement: An updated guideline for reporting systematic reviews. J. Clin. Epidemiol..

[44] Neiffer D.L., Hadfield C.A., Clayton L.A. (2021). Anesthesia and Analgesia. Clinical Guide to Fish Medicine.

[45] Esmail M.Y., Astrofsky K.M., Lawrence C., Serluca F.C., Fox J.G., Anderson L.C., Otto G.M., Pritchett-Corning K.R., Whary M.T. (2015). The Biology and Management of the Zebrafish. Laboratory Animal Medicine.

[46] Sneddon L.U. (2012). Clinical Anesthesia and Analgesia in Fish. J. Exot. Pet Med..

[47] Bramer W.M., Giustini D., de Jonge G.B., Holland L., Bekhuis T. (2016). De-duplication of database search results for systematic reviews in EndNote. J. Med. Libr. Assoc..

[48] McGrath S., Zhao X., Steele R., Thombs B.D., Benedetti A. (2020). Estimating the sample mean and standard deviation from commonly reported quantiles in meta-analysis. Stat. Methods Med. Res..

[49] Alestrom P., D’Angelo L., Midtlyng P.J., Schorderet D.F., Schulte-Merker S., Sohm F., Warner S. (2020). Zebrafish: Housing and husbandry recommendations. Lab. Anim..

[50] Zaig S., da Silveira Scarpellini C., Montandon G. (2021). Respiratory depression and analgesia by opioid drugs in freely behaving larval zebrafish. eLife.

[51] Lopez-Luna J., Al-Jubouri Q., Al-Nuaimy W., Sneddon L.U. (2017). Impact of stress, fear and anxiety on the nociceptive responses of larval zebrafish. PLoS ONE.

[52] Lopez-Luna J., Al-Jubouri Q., Al-Nuaimy W., Sneddon L.U. (2017). Reduction in activity by noxious chemical stimulation is ameliorated by immersion in analgesic drugs in zebrafish. J. Exp. Biol..

[53] Sneddon L.U. (2009). Pain Perception in Fish: Indicators and Endpoints. ILAR J..

[54] Ko M.J., Ganzen L.C., Coskun E., Mukadam A.A., Leung Y.F., van Rijn R.M. (2019). A critical evaluation of TRPA1-mediated locomotor behavior in zebrafish as a screening tool for novel anti-nociceptive drug discovery. Sci. Rep..

[55] Segner H. (2012). Fish. Nociception and Pain. A Biological Perspective.

[56] Guimaraes A.G., Quintans J.S., Quintans L.J. (2013). Monoterpenes with analgesic activity—A systematic review. Phytother. Res..

[57] Yang X., Wei X., Mu Y., Li Q., Liu J. (2020). A review of the mechanism of the central analgesic effect of lidocaine. Medicine.

[58] Stanley T.H. (2014). The fentanyl story. J. Pain.

[59] Rosland J.H., Tjølsen A., Bjørn M., Hole K. (1990). The formalin test in mice: Effect of formalin concentration. Pain.

[60] Sawynok J., Liu X.J. (2003). The Formalin Test: Characteristics and Usefulness of the Model. Rev. Analg..

[61] Hunskaar S., Hole K. (1987). The formalin test in mice: Dissociation between inflammatory and non-inflammatory pain. Pain.

[62] Oguri G., Nakajima T., Kikuchi H., Obi S., Nakamura F., Komuro I. (2021). Allyl isothiocyanate (AITC) activates nonselective cation currents in human cardiac fibroblasts: Possible involvement of TRPA1. Heliyon.

[63] Roberts A.C., Alzagatiti J.B., Ly D.T., Chornak J.M., Ma Y., Razee A., Zavradyan G., Khan U., Lewis J., Natarajan A. (2020). Induction of Short-Term Sensitization by an Aversive Chemical Stimulus in Zebrafish Larvae. eNeuro.

[64] Bao Y., Gao Y., Yang L., Kong X., Yu J., Hou W., Hua B. (2015). The mechanism of mu-opioid receptor (MOR)-TRPV1 crosstalk in TRPV1 activation involves morphine anti-nociception, tolerance and dependence. Channels.

[65] Stein C. (2018). New concepts in opioid analgesia. Expert Opin. Investig. Drugs.

[66] Webster L.R., Grabois M. (2015). Current Regulations Related to Opioid Prescribing. PM&R.

[67] Cashman J.N. (1996). The Mechanisms of Action of NSAIDs in Analgesia. Drugs.

[68] Munir M.A., Enany N., Zhang J.M. (2007). Nonopioid analgesics. Anesthesiol. Clin..

[69] Armstrong S.A., Herr M.J. (2024). Physiology, Nociception. StatPearls.

[70] Flecknell P.A. (1998). Analgesia in small mammals. Semin. Avian Exot. Pet Med..

[71] Hammer H.S., Cartner S.C., Eisen J.S., Farmer S.C., Guillemin K.J., Kent M.L., Sanders G.E. (2020). Water Quality for Zebrafish Culture. The Zebrafish in Biomedical Research.

[72] Lee C.J., Paull G.C., Tyler C.R. (2022). Improving zebrafish laboratory welfare and scientific research through understanding their natural history. Biol. Rev. Camb. Philos. Soc..

[73] Lawrence C. (2007). The husbandry of zebrafish (*Danio rerio*): A review. Aquaculture.

[74] Leal J.F., Neves M.G.P.M.S., Santos E.B.H., Esteves V.I. (2016). Use of formalin in intensive aquaculture: Properties, application and effects on fish and water quality. Rev. Aquac..

[75] Neiffer D.L., Stamper M.A. (2009). Fish Sedation, Anesthesia, Analgesia, and Euthanasia: Considerations, Methods, and Types of Drugs. ILAR J..

